# Thromboinflammation in COVID-19: Unraveling the interplay of coagulation and inflammation

**DOI:** 10.1097/MD.0000000000038922

**Published:** 2024-07-12

**Authors:** Emmanuel Ifeanyi Obeagu, Getrude Uzoma Obeagu

**Affiliations:** a Department of Medical Laboratory Science, Kampala International University, Kampala, Uganda; b School of Nursing Science, Kampala International University, Kampala, Uganda.

**Keywords:** coagulation, COVID-19, inflammation, thrombin

## Abstract

The coronavirus disease 2019 (COVID-19) pandemic has not only challenged global health systems but also spurred intense scientific inquiry into its pathophysiology. Among the multifaceted aspects of the disease, coagulation abnormalities have emerged as a significant contributor to morbidity and mortality. From endothelial dysfunction to dysregulated immune responses, various factors contribute to the hypercoagulable state seen in severe COVID-19 cases. The dysregulation of coagulation in COVID-19 extends beyond traditional thromboembolic events, encompassing a spectrum of abnormalities ranging from microvascular thrombosis to disseminated intravascular coagulation (DIC). Endothelial injury induced by severe acute respiratory syndrome coronavirus 2 (SARS-CoV-2) infection triggers a cascade of events involving platelet activation, coagulation factor consumption, and fibrinolysis impairment. Moreover, the virus direct effects on immune cells and the cytokine storm further exacerbate the prothrombotic milieu. Unraveling this intricate web of interactions between viral pathogenesis and host responses is essential for elucidating novel therapeutic targets and refining existing management strategies for COVID-19-associated coagulopathy. In the quest to unravel the complex interplay between coagulation and COVID-19, numerous clinical and laboratory studies have yielded invaluable insights into potential biomarkers, prognostic indicators, and therapeutic avenues. Anticoagulation therapy has emerged as a cornerstone in the management of severe COVID-19, although optimal dosing regimens and patient selection criteria remain subjects of ongoing investigation. Additionally, innovative approaches such as targeting specific components of the coagulation cascade or modulating endothelial function hold promise for future therapeutic development.

## 1. Introduction

The emergence of the novel coronavirus, severe acute respiratory syndrome coronavirus 2 (SARS-CoV-2), in late 2019 precipitated a global health crisis of unprecedented magnitude, culminating in the coronavirus disease 2019 (COVID-19) pandemic. As the virus spread rapidly across continents, infecting millions and claiming countless lives, scientists and healthcare professionals mobilized to understand its pathogenesis and develop effective strategies for containment and treatment. Amidst the myriad clinical manifestations of COVID-19, abnormalities in coagulation have emerged as a critical facet of the disease, significantly impacting patient outcomes and therapeutic management.^[1,2]^ COVID-19 encompasses a spectrum of clinical presentations, ranging from asymptomatic infection to severe respiratory distress syndrome and multiorgan failure. However, it became increasingly evident that beyond respiratory compromise, patients with severe disease often exhibited coagulation abnormalities characterized by thrombotic events and derangements in traditional coagulation parameters. Such observations raised intriguing questions regarding the underlying mechanisms driving coagulopathy in COVID-19 and its implications for disease progression and prognosis.^[3–5]^ Central to the pathogenesis of COVID-19-associated coagulopathy is the concept of endothelial dysfunction, wherein viral invasion of endothelial cells triggers a cascade of proinflammatory and procoagulant responses. SARS-CoV-2 gains entry into host cells via the angiotensin-converting enzyme 2 (ACE2) receptor, which is abundantly expressed on endothelial cells, leading to direct endothelial injury. Consequently, disrupted endothelial integrity contributes to platelet activation, leukocyte recruitment, and dysregulated release of von Willebrand factor (vWF), predisposing to microvascular thrombosis and macrovascular events. Furthermore, the ensuing inflammatory milieu characterized by cytokine release syndrome (CRS) amplifies endothelial dysfunction and coagulation activation, perpetuating a vicious cycle of thromboinflammation.^[6]^

Beyond endothelial injury, COVID-19-associated coagulopathy encompasses dysregulation of the coagulation cascade, fibrinolysis impairment, and consumption coagulopathy reminiscent of disseminated intravascular coagulation (DIC). Elevated levels of prothrombotic markers such as D-dimer, fibrinogen, and factor VIII underscore the hypercoagulable state observed in severe COVID-19 cases, posing significant challenges for clinical management. Moreover, the interplay between coagulation and inflammation extends beyond thrombotic events, influencing immune responses, tissue injury, and organ dysfunction in COVID-19. Deciphering the complex web of interactions between viral pathogenesis, host immune responses, and coagulation dynamics is essential for unraveling the pathophysiology of COVID-19-associated coagulopathy.^[7,8]^ Amidst the clinical challenges posed by COVID-19-associated coagulopathy, anticoagulation therapy has emerged as a cornerstone in the management of severe disease. Empirical use of prophylactic and therapeutic anticoagulation aims to mitigate thrombotic complications and improve patient outcomes, although optimal dosing strategies and patient selection criteria remain subjects of ongoing debate and investigation. Furthermore, the identification of novel biomarkers predictive of thrombotic risk and disease severity holds promise for refining risk stratification and guiding therapeutic interventions in COVID-19. Additionally, innovative therapeutic approaches targeting specific components of the coagulation cascade or modulating endothelial function represent fertile grounds for future research and therapeutic development.^[9]^

### 1.1. Aim

The aim of this study is to comprehensively elucidate the mechanisms of microvascular dysfunction in coagulation observed in COVID-19. Through a multidisciplinary approach encompassing clinical, translational, and basic science research, we aim to investigate the complex interplay between endothelial injury, dysregulated immune responses, and platelet activation in the pathogenesis of microvascular thrombosis and organ damage in severe COVID-19 cases. By understanding the underlying mechanisms driving microvascular dysfunction, we seek to identify potential therapeutic targets and guide the development of novel interventions aimed at mitigating thrombotic complications and improving patient outcomes in COVID-19. Through rigorous investigation and analysis, this study aims to contribute to the growing body of knowledge surrounding COVID-19-associated coagulopathy and provide insights that may inform clinical practice and public health strategies in the ongoing battle against the pandemic.

### 1.2. Rationale

The rationale for conducting this study stems from the pressing need to elucidate the intricate mechanisms underlying microvascular dysfunction in coagulation observed in COVID-19. Despite extensive research efforts, the pathophysiology of COVID-19-associated coagulopathy, particularly its impact on microvascular function, remains incompletely understood. Given the significant morbidity and mortality associated with thrombotic complications in severe COVID-19 cases, there is an urgent need to unravel the complexities of microvascular dysfunction to improve patient outcomes and guide therapeutic interventions. Microvascular dysfunction represents a critical aspect of COVID-19 pathogenesis, contributing to systemic thromboinflammation, organ damage, and multiorgan failure. However, the precise mechanisms driving microvascular thrombosis in COVID-19 remain poorly defined. By elucidating the underlying pathophysiological processes, including endothelial injury, dysregulated immune responses, and platelet activation, this study aims to provide insights into the molecular mechanisms driving microvascular dysfunction in COVID-19.

Furthermore, understanding the mechanisms of microvascular dysfunction may uncover potential therapeutic targets for intervention. Therapies targeting endothelial function, immune dysregulation, or platelet activation have the potential to mitigate thrombotic complications and improve outcomes in severe COVID-19 cases. Thus, by conducting a comprehensive investigation into the mechanisms of microvascular dysfunction, this study seeks to identify novel therapeutic strategies that could alleviate the burden of thrombotic complications and enhance the management of COVID-19. Moreover, by bridging the gap between clinical observations and basic science research, this study aims to contribute to the broader understanding of COVID-19 pathophysiology and coagulopathy. Insights gained from this study may inform clinical practice guidelines, risk stratification strategies, and therapeutic decision-making in the management of COVID-19 patients. Ultimately, by advancing our understanding of microvascular dysfunction in coagulation in COVID-19, this study has the potential to improve patient outcomes and mitigate the impact of thrombotic complications in this global health crisis.

## 2. Review methodology

### 2.1. Search strategy

A comprehensive search strategy was developed to identify relevant studies pertaining to microvascular dysfunction, coagulation, and COVID-19. Electronic databases including PubMed/MEDLINE, Embase, Scopus, and Web of Science were systematically searched for articles published up to the present date. The search terms included combinations of keywords such as “COVID-19,” “SARS-CoV-2,” “coagulation,” “thrombosis,” “microvascular dysfunction,” “endothelial injury,” “immune response,” and “platelet activation.”

### 2.2. Inclusion and exclusion criteria

Studies were included in the review if they investigated the mechanisms of microvascular dysfunction in coagulation observed in COVID-19, regardless of study design or publication type. Both clinical and basic science research articles were considered eligible for inclusion. Studies not directly relevant to the aim of the review or lacking sufficient data on microvascular dysfunction in COVID-19 were excluded.

### 2.3. Data extraction and synthesis

Data extraction was performed independently by 2 reviewers using a standardized data extraction form. Relevant information extracted from each study included study characteristics (e.g., authors, publication year), study design, participant characteristics, key findings related to microvascular dysfunction and coagulation in COVID-19, and any limitations identified by the reviewers. Discrepancies between reviewers were resolved through consensus or consultation with a third reviewer if necessary.

### 2.4. Quality assessment

The methodological quality and risk of bias of included studies were assessed using appropriate tools depending on the study design. For clinical studies, the Joanna Briggs Institute Critical Appraisal Checklist for Analytical Cross-Sectional Studies or Cohort Studies was used. For basic science studies, the Systematic Review Centre for Laboratory Animal Experimentation (SYRCLE) Risk of Bias Tool for animal studies or the National Institutes of Health (NIH) Quality Assessment Tool for preclinical and clinical studies was utilized.

### 2.5. Coagulation pathways and COVID-19

The coagulation pathways play a pivotal role in maintaining hemostasis, ensuring that blood remains in a fluid state under normal conditions while swiftly forming clots to prevent excessive bleeding upon vascular injury. However, in the context of COVID-19, dysregulation of coagulation pathways contributes to the pathogenesis of thrombotic complications observed in severe cases. The coagulation cascade comprises intrinsic and extrinsic pathways that converge at the activation of factor X, culminating in the conversion of prothrombin to thrombin and fibrinogen to fibrin, leading to clot formation. In COVID-19, endothelial injury triggered by SARS-CoV-2 infection disrupts the delicate balance of procoagulant and anticoagulant factors, tipping the scales towards a hypercoagulable state. Endothelial dysfunction promotes the release of vWF and tissue factor, initiating platelet activation and coagulation cascade activation, respectively. Consequently, increased thrombin generation and fibrin deposition contribute to microvascular thrombosis and macrovascular events seen in severe COVID-19 cases.^[10]^

Furthermore, the inflammatory response elicited by SARS-CoV-2 infection amplifies coagulation activation, perpetuating a cycle of thromboinflammation. CRS and the dysregulated immune response observed in severe COVID-19 exacerbate endothelial dysfunction and promote a prothrombotic milieu. Elevated levels of inflammatory cytokines such as interleukin-6 (IL-6) and tumor necrosis factor-alpha (TNF-α) further stimulate tissue factor expression and platelet activation, amplifying coagulation cascade activation and thrombin generation. Consequently, the crosstalk between inflammation and coagulation pathways contributes to the systemic thrombotic complications observed in COVID-19. Moreover, the dysregulation of fibrinolysis, the physiological process responsible for clot dissolution, further contributes to coagulopathy in COVID-19. Impaired fibrinolysis, characterized by elevated levels of plasminogen activator inhibitor-1 (PAI-1), inhibits the breakdown of fibrin clots, predisposing to thrombotic events and fibrin deposition in the microvasculature. Additionally, the formation of microthrombi in the pulmonary vasculature contributes to the pathogenesis of acute respiratory distress syndrome (ARDS) and respiratory failure, a hallmark of severe COVID-19. Thus, unraveling the intricate interplay between coagulation pathways, endothelial dysfunction, inflammation, and fibrinolysis is essential for understanding the pathophysiology of COVID-19-associated coagulopathy and guiding therapeutic interventions aimed at mitigating thrombotic complications and improving patient outcomes.^[10]^

### 2.6. Clinical implications of coagulation in COVID-19

The COVID-19 pandemic, caused by the novel coronavirus SARS-CoV-2, has brought to the forefront a complex and evolving understanding of the interaction between coagulation and the disease.^[11]^ Emerging evidence highlights that coagulation abnormalities play a pivotal role in the clinical course and outcomes of COVID-19 patients. In this section, we explore the clinical implications of coagulation disturbances in COVID-19, emphasizing their significant impact on patient health and prognosis.^[12]^ COVID-19 is associated with an increased risk of venous thromboembolism, including deep vein thrombosis (DVT) and pulmonary embolism (PE). Arterial thrombosis, such as myocardial infarction and stroke, is also observed in COVID-19 patients. These thrombotic events can lead to serious complications and significantly affect patient outcomes.^[13,14]^ COVID-19 can trigger DIC; a condition characterized by widespread clot formation throughout the body. DIC can result in excessive bleeding, multi-organ failure, and a poor prognosis.^[15]^ Microvascular thrombosis, including capillary plugging, is a prominent feature in severe COVID-19 cases. This microvascular dysfunction contributes to organ damage, particularly in the lungs and kidneys, affecting oxygen exchange and filtration.^[16]^ The coagulation disturbances in COVID-19, often leading to thrombotic events, can result in organ failure. Patients may experience acute respiratory distress syndrome (ARDS), myocardial injury, acute kidney injury, and other complications.^[17]^

Coagulation markers, such as elevated D-dimer levels, are associated with worse outcomes in COVID-19. Patients with severe coagulation abnormalities are more likely to require intensive care, mechanical ventilation, and have a higher mortality rate.^[18]^ Risk stratification based on coagulation markers and clinical assessment may aid in identifying COVID-19 patients at higher risk of thrombotic complications. Tailored interventions can be applied to these high-risk individuals to mitigate adverse outcomes.^[19]^ The clinical implications of coagulation disturbances have led to adjustments in treatment strategies. Anticoagulant therapy, such as prophylactic and therapeutic anticoagulation, is increasingly utilized to prevent and treat thrombotic events in COVID-19 patients.^[20]^ Close monitoring of coagulation markers and clinical status is essential for managing COVID-19 patients. Ongoing assessment and follow-up are crucial to adapt treatment as the clinical course evolves.^[21]^ The clinical implications of coagulation abnormalities in COVID-19 are profound and far-reaching. Thrombotic events, DIC, microvascular dysfunction, and organ failure are among the critical outcomes associated with coagulation disturbances.^[22,23]^ Recognizing the impact of coagulation on patient prognosis has led to adjustments in treatment strategies and the utilization of anticoagulants to mitigate these adverse clinical implications. A comprehensive understanding of these coagulation-related clinical implications is vital for improving the management and outcomes of COVID-19 patients.

### 2.7. Mechanisms of microvascular dysfunction in coagulation in COVID-19

Microvascular dysfunction is a critical component of coagulation abnormalities observed in COVID-19, contributing to the pathogenesis of thrombotic complications and organ damage. Several mechanisms underlie microvascular dysfunction in the context of COVID-19, including endothelial injury, dysregulated immune responses, and platelet activation. Endothelial injury lies at the crux of microvascular dysfunction in COVID-19. SARS-CoV-2 gains entry into host cells via the ACE2 receptor, which is abundantly expressed in endothelial cells. Viral invasion triggers direct endothelial injury, disrupting endothelial integrity and activating proinflammatory and procoagulant pathways. Consequently, dysfunctional endothelium promotes platelet adhesion, leukocyte recruitment, and dysregulated release of vWF, leading to microvascular thrombosis and impaired blood flow.^[24–28]^

Dysregulated immune responses further exacerbate microvascular dysfunction in COVID-19. The cytokine storm observed in severe cases leads to systemic inflammation and endothelial activation, perpetuating endothelial injury and thromboinflammation. Inflammatory cytokines such as IL-6 and TNF-α upregulate adhesion molecules and tissue factor expression on endothelial cells, promoting coagulation cascade activation and thrombus formation in the microvasculature. Additionally, activated immune cells release reactive oxygen species and proinflammatory mediators, further compromising endothelial function and perpetuating microvascular dysfunction.^[29–31]^ Platelet activation plays a pivotal role in microvascular dysfunction and thrombotic complications in COVID-19. Endothelial injury and systemic inflammation trigger platelet activation, leading to the release of prothrombotic factors and amplifying coagulation cascade activation. Moreover, platelets interact with leukocytes and endothelial cells, forming platelet-leukocyte aggregates and promoting endothelial activation and thrombus formation. The dysregulated cross-talk between platelets, leukocytes, and endothelial cells contributes to microvascular thrombosis and organ damage in severe COVID-19.^[32–34]^

### 2.8. Therapeutic approaches for coagulation in COVID-19

The recognition of coagulation abnormalities in COVID-19 has prompted extensive research into therapeutic strategies aimed at mitigating the prothrombotic aspects of the disease. The complex interaction between SARS-CoV-2 and the coagulation system has led to a range of potential treatment approaches.^[35,36]^ Anticoagulants, such as heparin and low molecular weight heparin (LMWH), are commonly used to prevent and treat thrombotic events in COVID-19 patients. Both prophylactic and therapeutic anticoagulation strategies have been employed, with dosing adjusted based on the patient clinical condition and coagulation markers.^[37]^ Antiplatelet drugs, including aspirin and P2Y12 receptor antagonists, are being explored for their potential to inhibit platelet activation and aggregation. Reducing platelet involvement in clot formation may help mitigate thrombotic complications.^[38]^ In some severe COVID-19 cases, where large thrombi obstruct vital blood vessels, fibrinolytic therapy with drugs like tissue plasminogen activator (tPA) may be considered. Fibrinolytic therapy aims to dissolve existing clots and restore blood flow.^[39]^ Direct thrombin inhibitors, such as argatroban and bivalirudin, can block the activity of thrombin, a key enzyme in the coagulation cascade. These agents are being studied for their potential to mitigate thrombosis in COVID-19.^[40]^ Certain immunomodulatory therapies, such as corticosteroids and intravenous immunoglobulin (IVIG), have been shown to reduce inflammation and, indirectly, coagulation abnormalities. These therapies are primarily used in severe cases with hyperinflammatory responses.^[41]^ Stabilizing the endothelial cells lining blood vessels is a potential strategy to reduce activation and subsequent clot formation. Statins and agents that promote vascular endothelial function are under investigation.^[42]^ Given the role of complement activation in promoting coagulation, complement inhibitors are being explored. These inhibitors may help modulate the prothrombotic response.^[43]^ Plasma exchange therapy aims to remove inflammatory mediators and coagulation factors from the patient blood. It may be considered in cases of severe cytokine storm and coagulation abnormalities.^[44]^ The approach to treatment is increasingly personalized, with dosing and interventions adjusted based on individual patient characteristics and coagulation profiles. This tailored approach considers the delicate balance between preventing thrombosis and avoiding bleeding complications.^[45]^ Ongoing clinical trials are evaluating the effectiveness of various therapeutic approaches, providing valuable data for evidence-based management. The management of coagulation abnormalities in COVID-19 is complex, requiring a multidisciplinary approach that considers the patient clinical condition, coagulation markers, and individual risk factors. As our understanding of the disease and its interactions with the coagulation system continues to evolve, the development of targeted therapeutic interventions offers hope for improving patient outcomes and reducing the thrombotic complications associated with COVID-19.^[46]^

### 2.9. Challenges and future directions of coagulation and COVID-19

Coagulation and its association with COVID-19 have been areas of significant interest and research since the beginning of the COVID-19 pandemic.^[47]^ COVID-19 has been associated with an increased risk of coagulation abnormalities, which can have serious clinical implications. Here are some of the challenges and future directions related to coagulation and COVID-19:

## 3. Challenges

COVID-19 patients are at an increased risk of developing thrombosis, which can lead to serious complications such as DVT, PE, and stroke. Managing this risk is a significant challenge, and the optimal strategies for anticoagulation in COVID-19 patients are still a subject of ongoing research.^[48]^ Balancing the need for anticoagulation to prevent thrombosis with the risk of bleeding in COVID-19 patients can be challenging, especially in critically ill patients. Clinicians need to make individualized decisions, and more research is needed to guide these decisions.^[49]^ Understanding the precise mechanisms underlying COVID-19-related coagulopathy is still an ongoing challenge. While there evidence of endothelial damage and inflammation playing a role, further research is needed to unravel the complex interactions.^[50]^ It still unclear how COVID-19-related coagulation abnormalities may impact patients in the long term. Some patients experience prolonged symptoms, and it essential to study the potential long-term consequences of coagulation issues.^[51]^

## 4. Future directions

Future research may focus on identifying biomarkers or genetic factors that can help predict which COVID-19 patients are at the highest risk of coagulation issues. This would enable more personalized treatment approaches.^[52]^ Continued research is needed to determine the most effective and safe anticoagulation strategies for COVID-19 patients, taking into account individual risk factors and disease severity.^[53]^ There is a need for standardized guidelines for thromboprophylaxis in COVID-19 patients. Ongoing research will help define the appropriate dosing and duration of anticoagulant therapy for various patient groups.^[46]^ Studying the long-term outcomes of COVID-19 patients with coagulation abnormalities is crucial. This will help us understand the potential for chronic health issues and inform follow-up care.^[54]^ Research into new treatments, such as targeting specific components of the coagulation cascade or endothelial function, may yield novel therapeutic options for COVID-19 patients with coagulation issues.^[55]^ As COVID-19 vaccines are administered globally, monitoring and researching potential coagulation issues related to vaccination, like vaccine-induced immune thrombotic thrombocytopenia (VITT), is another important future direction.^[56]^ The relationship between coagulation and COVID-19 is a complex and evolving field of study. Researchers and healthcare providers continue to work towards a better understanding of the mechanisms and appropriate management strategies for coagulation abnormalities in COVID-19 patients. This knowledge is essential for improving patient outcomes and reducing the burden of thrombotic complications associated with the disease.^[57]^

## 5. Products formed in the coagulation pathways of COVID-19 patients

In COVID-19 patients, the coagulation pathways often undergo dysregulation, leading to various products and factors that contribute to abnormal clotting tendencies.^[58]^ The activation of the coagulation cascade and inflammatory responses can result in the formation of several products:

Fibrin Clots: Elevated levels of fibrinogen, a plasma protein, lead to the production of fibrin, resulting in the formation of blood clots. Excessive fibrin formation can contribute to microthrombi in blood vessels, affecting various organs.

D-dimer: Increased levels of D-dimer, a breakdown product of cross-linked fibrin, are commonly observed in severe COVID-19 cases. Elevated D-dimer levels indicate active fibrinolysis and the breakdown of blood clots.

Platelet Activation: COVID-19 can induce platelet activation and aggregation. Activated platelets contribute to the formation of thrombi and exacerbate the procoagulant state.^[58]^

vWF: vWF is involved in platelet adhesion and is elevated in COVID-19. Increased vWF levels contribute to platelet activation and clot formation.

Tissue Factor (TF): TF, a protein involved in initiating the coagulation cascade, can be upregulated in response to inflammation in COVID-19 patients, leading to excessive activation of clotting pathways.

Procoagulant Factors: Factors such as Factor VIII and Factor IX, essential in the coagulation cascade, may be elevated in severe COVID-19, contributing to a procoagulant state.^[58]^

Anticoagulant Pathway Alterations: Disruption of the anticoagulant pathways, including reduced levels of antithrombin and protein C, can impair the body ability to regulate clotting, increasing the risk of thrombosis.

Endothelial Dysfunction Products: COVID-19 can cause endothelial damage, resulting in the release of factors that promote coagulation and vasoconstriction, exacerbating clot formation.^[58]^

These products and factors formed in the coagulation pathways of COVID-19 patients contribute to a hypercoagulable state, leading to an increased risk of thrombosis and complications such as PE, strokes, myocardial infarctions, and organ damage.^[59]^ Monitoring these coagulation products helps healthcare professionals assess the severity of the coagulopathy and guide appropriate therapeutic interventions, including anticoagulation therapy, to manage the coagulation abnormalities and reduce the risk of thrombotic events in COVID-19 patients. Table 1 shows major coagulation disorders in COVID-19 Patients (provided by authors).

**Table 1:** Major coagulation disorders in COVID-19 patients.

Group	Name	Abbreviation	Dominant changes
Coagulation problems	Arterial thromboembolism	ATE	Blood clotting
	Deep vein thrombosis	DVT	Blood clotting
	Disseminated intravascular coagulation/sepsis-induced coagulopathy	DIC/SIC	Blood clotting
	Pulmonary embolism	PE	Blood clotting
	Venous thromboembolism	VT	Blood clotting
Coagulation parameters	D-dimer	Dd	Increase
	Factor VIII/Von Willebrand factor	FVIII/VWF	Increase
	Fibrinogen and Fibrin degradation products	FDP	Increase
	Lymphocyte count	PL	Decrease
	Partial thromboplastin time/Prothrombin time	PTT/PT	Get longer
	Platelet count	PC	Decrease

COVID-19 = coronavirus disease 2019.^[59]^

## 6. Policy recommendations

1. Enhanced Surveillance and Monitoring: Implement robust surveillance systems to monitor thrombotic events and microvascular dysfunction in COVID-19 patients. Develop standardized protocols for the timely detection and management of thrombotic complications, including routine screening for biomarkers of coagulopathy such as D-dimer and fibrinogen levels.
2. Early Intervention and Risk Stratification: Develop risk stratification tools to identify COVID-19 patients at higher risk of developing thrombotic complications and microvascular dysfunction. Implement early intervention strategies, including prophylactic anticoagulation, in high-risk patients to mitigate the risk of thrombosis and improve clinical outcomes.
3. Optimized Anticoagulation Therapy: Establish evidence-based guidelines for the use of anticoagulation therapy in COVID-19 patients. Tailor anticoagulant regimens based on individual patient characteristics, disease severity, and risk factors for thrombosis. Consider novel anticoagulants or combination therapies to optimize thromboprophylaxis in severe COVID-19 cases.
4. Multidisciplinary Care Teams: Foster collaboration between healthcare professionals from diverse specialties, including pulmonology, hematology, critical care, and infectious diseases, to provide comprehensive care for COVID-19 patients with thrombotic complications. Implement multidisciplinary care teams to facilitate early detection, prompt intervention, and coordinated management of microvascular dysfunction in COVID-19.
5. Patient Education and Empowerment: Empower patients and caregivers with information about the signs and symptoms of thrombotic complications and the importance of adherence to anticoagulation therapy. Provide educational materials and resources to enhance patient understanding of the risks associated with COVID-19-associated coagulopathy and the importance of early medical intervention.
6. Research and Innovation: Invest in research to further elucidate the underlying mechanisms of microvascular dysfunction in coagulation in COVID-19 and identify novel therapeutic targets. Support innovative research initiatives aimed at developing targeted interventions to prevent and treat thrombotic complications in COVID-19 patients, including the use of endothelial-protective agents, anti-inflammatory therapies, and novel anticoagulants.
7. Global Collaboration and Knowledge Sharing: Facilitate international collaboration and knowledge sharing among researchers, healthcare professionals, and policymakers to address the challenges posed by microvascular dysfunction in coagulation in COVID-19. Foster partnerships between academia, industry, and government agencies to accelerate the development and implementation of effective strategies for the prevention and management of thrombotic complications in COVID-19 patients worldwide.
8. Public Health Preparedness: Strengthen public health infrastructure and preparedness measures to respond effectively to future pandemics and emerging infectious diseases. Develop contingency plans and resource allocation strategies to ensure timely access to critical care services, medical supplies, and medications for the management of COVID-19-associated coagulopathy and other complications.

## 7. Conclusion

The review of the mechanisms underlying microvascular dysfunction in coagulation in COVID-19 underscores the complexity of this phenomenon and its significant implications for patient outcomes. Through a systematic analysis of the literature, we have gained valuable insights into the interplay between endothelial injury, dysregulated immune responses, and platelet activation in the pathogenesis of microvascular thrombosis and organ damage in severe COVID-19 cases. The findings highlight the multifactorial nature of COVID-19-associated coagulopathy and underscore the need for a multidisciplinary approach to its management. Enhanced surveillance, early intervention, and optimized anticoagulation therapy are essential components of a comprehensive approach to addressing microvascular dysfunction in COVID-19. Moreover, fostering collaboration between healthcare professionals, investing in research and innovation, and strengthening public health preparedness are critical steps towards mitigating the impact of microvascular dysfunction on patient outcomes and improving the management of COVID-19. The elucidation of the mechanisms of microvascular dysfunction in coagulation in COVID-19 represents a crucial step towards advancing our understanding of the pathophysiology of the disease and guiding therapeutic interventions. By implementing evidence-based policies and strategies informed by these findings, we can enhance patient care, reduce the burden of thrombotic complications, and ultimately improve outcomes for individuals affected by COVID-19.

## Author contributions

**Conceptualization:** Emmanuel Ifeanyi Obeagu.

**Methodology:** Emmanuel Ifeanyi Obeagu, Getrude Uzoma Obeagu.

**Supervision:** Emmanuel Ifeanyi Obeagu.

**Validation:** Emmanuel Ifeanyi Obeagu.

**Visualization:** Emmanuel Ifeanyi Obeagu.

**Writing – original draft:** Emmanuel Ifeanyi Obeagu, Getrude Uzoma Obeagu.

**Writing – review & editing:** Emmanuel Ifeanyi Obeagu, Getrude Uzoma Obeagu.
